# Effects of abdominal visceral fat compared with those of subcutaneous fat on the association between PM_10_ and hypertension in Korean men: A cross-sectional study

**DOI:** 10.1038/s41598-019-42398-1

**Published:** 2019-04-11

**Authors:** Hyun-Jin Kim, Hyuktae Kwon, Su-Min Jeong, Seo Eun Hwang, Jin-Ho Park

**Affiliations:** 1 National Cancer Control Institute, National Cancer Center, Goyang, 10408 South Korea; 2 Department of Family Medicine, Seoul National University Hospital, Seoul, 03080 South Korea; 3 Department of Family Medicine, Seoul National University College of Medicine, Seoul, 03080 South Korea

## Abstract

We assessed whether visceral adipose tissue (VAT) compared with subcutaneous adipose tissue (SAT) has modifying effects on the cross-sectional association between ambient air pollution and hypertension in Korean men. This study included 1,417 adult men who visited a health checkup center. Abdominal fat depots were measured by computed tomography, and we used the annual average concentrations of ambient air pollutants such as particulate matter with an aerodynamic diameter of ≤10 μm (PM_10_), nitrogen dioxide, sulfur dioxide, and carbon monoxide (CO). The annual mean concentrations of PM_10_ (odds ratio [*OR*] = 1.30; 95% confidence interval [*CI*] = 1.12–1.52) and CO (OR = 1.20; 95% CI = 1.03–1.39) showed a positive association with hypertension. In particular, modifying effects on hypertension were found between PM_10_ and VAT-related traits such as VAT and visceral-to-subcutaneous fat ratio (VSR). The association between PM_10_ and hypertension was much stronger in the high-VAT (*OR* = 1.74; 95% *CI* = 1.12–2.71) and high-VSR groups (*OR* = 1.53; 95% *CI* = 1.23–1.91). However, the strength of association across levels of SAT was not observed (*P*_int_ = 0.4615). In conclusion, we found that association between PM_10_ exposure and hypertension is different by abdominal fat distribution.

## Introduction

Ambient air pollution, including particulate matter (PM), is a serious health burden worldwide and has recently emerged as the biggest social issue in the Korean society^1^. The number of deaths from air pollution is estimated at approximately 3 million worldwide each year^2^. A recent report from the Organization for Economic Cooperation and Development (OECD) documented that Korea will have the most significant increase in the number of premature deaths caused by outdoor air pollution among the OECD countries by 2060^3^. The majority of air pollution-related premature deaths are associated with cardiovascular events, such as myocardial infarction, stroke, and heart attack, and hypertension is regarded as a crucial risk factor in the development of such cardiovascular disease^4,5^. In recent years, a growing number of epidemiological studies have identified the deleterious effects of ambient air pollution on hypertension^6–8^.

More recent evidence has suggested the importance of obesity in association between long-term air pollution exposure and blood pressure (BP) increments^9–11^. Although the biological pathways underlying this connection have not been completely elucidated, several plausible hypotheses, such as inflammatory and oxidative stress, have been proposed. However, given that central adiposity is a stronger marker for major cardiovascular diseases compared with simple obesity markers, such as body mass index (BMI), and given that its regional distribution, such as subcutaneous and visceral adiposity, plays different roles in the development of such diseases, the depot-specific characteristics of visceral and subcutaneous fat in obesity need to be distinguished to understand these mechanisms. Excessive accumulation of visceral adipose tissue (VAT) has been shown to contribute to the development of cardiovascular diseases via the activation of inflammatory molecules or systemic oxidative damage, even in people with a normal BMI^12–14^. VAT produces more proinflammatory cytokines, such as interleukin-6 (IL-6), tumor necrosis factor-α (TNF-α), and visfatin, than subcutaneous adipose tissue (SAT), and this triggers the overproduction of reactive oxygen species (ROS)^15,16^. In contrast, the subcutaneous fat compartments might even play a protective role in health outcomes^16^. Within this context, the modification effect of obesity on the association between air pollution and hypertension may be differentiated by adiposity traits; therefore, studies using the accurate measurement of fat mass measured by computed tomography (CT) are needed. However, to the best our knowledge, no studies have been conducted to evaluate such a hypothesis regarding regional fat distributions.

This study aimed to investigate the cross-sectional association between ambient air pollution and hypertension in Korean men and to identify whether these relations are modified by abdominal fat distribution, especially visceral fat.

## Materials and Methods

### Participants

The subjects in this study were recruited from a health checkup center managed by Seoul National University Hospital from 2006 to 2014^17,18^. Briefly, In South Korea, adults in the general population usually receive regular comprehensive health screenings for the prevention or early detection of the disease. For this reason, large hospitals in South Korea operate comprehensive health check-up center for them. Abdominal fat distribution is very different by sex, and Korean men are known to have a larger distribution of visceral fat than women. In fact, when we classified into three groups by adiposity level using the identical cut-off points in men and women, the sample size of high VAT (n = 26) or visceral-to-subcutaneous fat ratio (VSR) (n = 48) group in women was a very small for statistical analysis (Data not shown). Thus, only male subjects were considered for the study. A total of 1,759 adult men were enrolled during this period, and 342 subjects without abdominal CT data were excluded from this study. Therefore, a total of 1,417 subjects were included in the final statistical analysis. Considering the purpose of this study to investigate the relationship between air pollution and hypertension is modified by abdominal fat, it was inevitable to exclude subjects without abdominal CT information. There was no big difference between the included (n = 1,417) and excluded (n = 342) samples in BP traits and hypertension. In addition, the distributions of BMI and anthropometric data including height and weight were similar between included and excluded samples. In case of age, the distribution between the included and excluded samples was also similar. However, there were a little difference between the two samples in lifestyle such as smoking status, drinking, and physical activity (Data not shown). The protocol for this study was approved by the institutional review board of the Seoul National University Hospital Biomedical Research Institute (IRB no.: 1708-017-873), and all participants provided informed written consent before the initiation of the study. All methods were performed in accordance with the relevant guidelines and regulations.

### Assessment of hypertension

All the evaluation including anthropometric, BP, and laboratory measurement were done in 12 hours overnight fasting state. In case of BP measurement, each subject was requested to sit quietly in a chair with back supported at least for 5 minutes before measurement is taken. All the BP measurements were done using the same validated automatic BP device (EASY-X800 model from JAWON Medical). Two separate measurements with minimum 1 minute interval were taken. The average value of two readings was taken as each subject’s final systolic blood pressure (SBP) and diastolic blood pressure (DBP) data. We also checked whether each individual was taking antihypertensive medications or not. Hypertension was defined as a SBP of ≥140 mmHg, a DBP of ≥90 mmHg, or taking antihypertensive medications. In addition, for subjects who were taking antihypertensive medications, we added 10 mmHg and 5 mmHg to the measured SBP and DBP values, respectively, before analysis for continuous outcomes such as SBP and DBP^19^.

### Assessment of obesity/abdominal adiposity

Based on the Asia-Pacific obesity classification of adult Asians^20^, the subjects were classified into three groups: underweight or normal (BMI <23 kg/m^2^), overweight (23 kg/m^2^ ≤ BMI <25 kg/m^2^), and obese (BMI ≥25 kg/m^2^). In addition, abdominal adiposity was measured using the Somatom Sensation 16 CT scanner (Siemens AG, Erlangen, Germany). We estimated the cross-sectional area of abdominal fat compartments using Rapidia 2.8 software (from −250 to −50 Hounsfield units] (INFINITT, Seoul, Korea). The VAT area was defined by delineating intra-abdominal fat bound by parietal peritoneum or transversalis fascia, excluding the vertebra and spinal muscles. SAT area was defined as fat tissue located between inside of dermis and outside of back and abdominal muscles. The VSR was also calculated. We considered the first cut-off value for VAT at 100 cm^2^, which has been reported as a reasonable criterion for screening for obesity-related cardiovascular disorders^21,22^. In addition, to define the high visceral obesity group, the second cut-off point for VAT was applied at 200 cm^2^, twice the screening cut-off level (VAT = 100 cm^2^)^23^. Therefore, we classified into three groups according to VAT level: low-VAT group (VAT ≤ 100 cm^2^), intermediate VAT group (100 cm^2^ < VAT ≤ 200 cm^2^), and high-VAT group (VAT > 200 cm^2^). Subjects were also categorized into three subgroups according SAT level using the same criteria as VAT, because of the absence of optimal cut-off criterion in an Asian population for SAT: low-SAT group (SAT ≤ 100 cm^2^), intermediate SAT group (100 cm^2^ < SAT ≤ 200 cm^2^), and high-SAT group (SAT >200 cm^2^). In addition, we divided into three groups according to VSR level: low-VSR group (VSR ≤ 0.8), intermediate VSR group (0.8 < VSR ≤ 1.0), and high-VSR group (VSR > 1.0).

### Covariate variables

To control the effects of potential confounding variables, we examined the health-related behaviors such as smoking status and alcohol drinking. These items were investigated using a structured questionnaire and were categorized as follows: smoking status (never, former, or current smoker) and alcohol drinking (never, former, or current drinker). Physical activity was defined as moderate- or vigorous-intensity physical activities engagement of more than 10 min at a time at least one day per week. This variable was coded as a binary variable (“yes” or “no”). To identify the potential covariate variable for hypertension, a univariate analysis was performed (Table S1). First, the variables such as age, BMI, and smoking status, which are related to hypertension, were considered as covariates. Second, we also adjusted the physical activity and alcohol consumption, which is essentially considered as covariate variables for hypertension studies, based on previous literature^6,8,9^, even though their associations were not observed in our data (Table S1).

### Assessment of air pollution exposure

We used atmospheric monitoring data for 24-h concentrations of ambient air pollutants from the Ministry of the Environment of Korea (https://www.airkorea.or.kr). The data included concentrations of air pollutants, such as PM with an aerodynamic diameter of ≤10 μm (PM_10_), nitrogen dioxide (NO_2_), sulfur dioxide (SO_2_), and carbon monoxide (CO), measured in approximately 300 national monitoring stations from January 1, 2006 to December 31, 2014. In addition, each subject’s residential zip code information was obtained from the hospital information system database. Using this zip code information, we identified the nearest monitoring station to each subject’s home. To assess the exposure concentrations to air pollutants, we calculated the annual average concentrations corresponding to the health checkup year at the closest monitoring station from each subject’s residence. All four air pollutants are interrelated, with correlation ranging from 0.21 to 0.61 (Table S2). To perform the subgroup analysis stratified by PM_10_ levels, the quantitative PM_10_ exposure was classified into four levels using quartiles: quartile 1 (PM_10_ ≤ 43.8 mg/m^3^), quartile 2 (43.8 mg/m^3^ < PM_10_ ≤ 48.7 mg/m^3^), quartile 3 (48.7 mg/m^3^ < PM_10_ ≤ 55.0 mg/m^3^), and quartile 4 (PM_10_ > 55.0 mg/m^3^). For the final analysis, we divided the subjects into three PM_10_ exposure groups using these quartiles: low exposure (quartile 1), intermediate exposure (quartiles 2–3), and high exposure (quartile 4).

### Statistical analysis

The associations between adiposity trait or ambient air pollution and hypertension were determined by multiple logistic regression analysis. The odds ratios (ORs) and 95% confidence intervals (CIs) of adiposity trait or air pollution for hypertension were estimated in unadjusted and adjusted models for covariates such as age, smoking status, alcohol consumption, and physical activity. In addition, we performed multiple linear regression analysis to identify associations between adiposity trait or ambient air pollution and quantitative BP trait (i.e. SBP or DBP). The beta coefficients (*β*) and standard errors (*SE*) of adiposity trait or air pollution for BP traits were estimated in unadjusted and adjusted models for covariates such as age, smoking status, alcohol consumption, and physical activity. The estimates in adiposity traits (i.e. VAT and SAT) and air pollutants were converted by scale to the 100-cm^2^ area and interquartile range (IQR) for each air pollutant (11.2 μg/m^3^ for PM_10_, 15.6 ppb for NO_2_, 1.9 ppb for SO_2_, and 2.0 ppm for CO) (Table S2). We also performed a stratified analysis for groups with three different levels of abdominal adiposity trait such as BMI, VAT, SAT, and VSR. In addition, we applied the multiple regression approach with interaction term to test the modifying effect of adiposity level. The modifications on BP traits and hypertension were identified by performing multiple linear regression analysis [*Y* = *β*_0_ + *β*_1_age + *β*_2_smoking status + *β*_3_alcohol consumption + *β*_4_physical activity + *β*_5_adiposity level + *β*_6_air pollution exposure + *β*_7_(adiposity level × air pollution exposure) + *e*] and multiple logistic regression analysis [logit(*P*) = *β*_0_ + *β*_1_age + *β*_2_smoking status + *β*_3_alcohol consumption + *β*_4_physical activity + *β*_5_adiposity level + *β*_6_air pollution exposure + *β*_7_(adiposity level × air pollution exposure) + *e*], respectively. Using the same method as stratification analysis by the adiposity level, we also have performed the subgroup analysis stratified by PM_10_ levels. All analyses were performed using SAS 9.3 version (SAS Institute, Cary, NC, USA).

## Results

### The characteristics of the study subjects

Table 1 shows the detailed characteristics of our study subjects. A total of 1,417 men were included in the final association analyses. All subjects were men, and their mean age was 55.9 years. The percentage of current smokers and current drinkers were 31.3% (n = 444) and 66.5% (n = 942), respectively. The mean BMI value was 24.6 kg/m^2^. With regard to adiposity trait, the mean value of VAT (133.0 cm^2^) was similar to that of SAT (136.4 cm^2^), and the mean VSR value was 1.0. Mean SBP and DBP values were 127.6 mmHg and 77.5 mmHg, respectively, and about 27% of the subjects were taking antihypertensive medications. Ultimately, 41.9% of the subjects were classified as hypertensive patients (n = 593).

**Table 1:** Characteristics of study participants.

Characteristics	n(%) or mean (SD)
n	1,417
Age (years)	55.9 (9.3)
**Smoking**	
Never	309 (21.8)
Former-smokers	664 (46.9)
Current-smokers	444 (31.3)
**Alcohol drinking**	
Never	354 (25.0)
Former-drinkers	121 (8.5)
Current- drinkers	942 (66.5)
**Physical activity**	
Yes	532 (37.5)
No	885 (62.5)
Height (cm)	169.0 (6.1)
Weight (kg)	70.2 (9.7)
BMI(kg/m^2^)	24.6 (2.9)
**Adiposity measures**	
VAT (cm^2^)	133.0 (59.0)
SAT (cm^2^)	136.4 (55.0)
VSR	1.0 (0.4)
**Blood pressure**	
SBP (mmHg)	127.6 (14.8)
DBP(mmHg)	77.5 (10.7)
**Antihypertensive medication**	
Yes	377 (26.6)
No	1,040 (73.4)
**Hypertension**	
Yes	593 (41.9)
No	824 (58.1)

BMI, body mass index; VAT, visceral adipose tissue; SAT, subcutaneous adipose tissue; VSR, visceral-to-subcutaneous fat ratio; SBP, systolic blood pressure; DBP, diastolic blood pressure

Data are presented as mean (standard deviation) for continuous variables, or n (%) for categorical variables.

### The association between adiposity-related traits or air pollutants and hypertension

We investigated the association between BP traits and risk factors (i.e. adiposity-related traits and air pollutants) before evaluating hypertension (Table S3). All adiposity-related traits were positively associated with both SBP and DBP. In the case of air pollution, only PM_10_ in the adjustment model showed a positive association with both SBP (*β* = 2.13; *SE* = 0.58) and DBP (*β* = 1.33; *SE* = 0.41). In addition, IQRs increases in NO_2_ (*β* = 1.16; *SE* = 0.55) and CO (*β* = 1.26; *SE* = 0.60) concentrations in adjusted model were related to increase of SBP. Logistic regression analysis results of the association between adiposity-related traits or air pollutants and hypertension are shown in Table 2. In both unadjusted and adjusted models, all adiposity-related traits, including BMI, VAT, SAT, and VSR, were positively associated with hypertension. The OR of VAT area per 100 cm^2^ increase (adjusted model: *OR* = 1.98; 95% *CI* = 1.63–2.41) was slightly higher than that of the SAT area per 100 cm^2^ increase (adjusted model: *OR* = 1.84; 95% *CI* = 1.49–2.27). With regard to the association between air pollution and hypertension, an IQR (11.2 μg/m^3^) increase in PM_10_ concentrations was positively associated with hypertension (adjusted model: *OR* = 1.30; 95% *CI* = 1.12–1.52). An IQR (0.2 ppm) increase in CO concentrations also showed a positive association with hypertension (adjusted model: *OR* = 1.20; 95% *CI* = 1.03–1.39). However, NO_2_ (adjusted model: *OR* = 1.07; 95% *CI* = 0.93–1.24) and SO_2_ (adjusted model: *OR* = 1.01; 95% *CI* = 0.89–1.15) did not show an association with hypertension.

**Table 2:** Logistic regression results for the association between air pollution, adiposity-related traits, and hypertension

	Hypertension			
	Unadjusted Model		Adjusted Model	
	*OR* (95% CI)	*p-*value	*OR* (95% CI)	*p-*value
**Adiposity trait**				
BMI (kg/m^2^)	1.14 (1.10–1.18)	<0.0001	1.17 (1.13–1.22)	<0.0001
VAT (cm^2^)	2.06 (1.70–2.49)	<0.0001	1.98 (1.63–2.41)	<0.0001
SAT (cm^2^)	1.61 (1.33–1.97)	<0.0001	1.84 (1.49–2.27)	<0.0001
VSR	1.58 (1.22–2.04)	0.0005	1.31 (1.00–1.71)	0.0446
**Air pollution**				
PM_10_ (μg/m^3^)	1.24 (1.08–1.43)	0.0028	1.30 (1.12–1.52)	0.0005
NO_2_ (ppb)	1.07 (0.93–1.23)	0.3311	1.07 (0.93–1.24)	0.3191
SO_2_ (ppb)	1.00 (0.88–1.14)	0.9868	1.01 (0.89–1.15)	0.8606
CO (ppm)	1.18 (1.02–1.37)	0.0303	1.20 (1.03–1.39)	0.0230

BMI, Body mass index; VAT, visceral adipose tissue; SAT, subcutaneous adipose tissue; VSR, visceral-to-subcutaneous fat ratio; PM_10_, particulate matter ≤ 10 μm in diameter; NO_2_, nitrogen dioxide; SO_2_, sulfur dioxide; CO, carbon monoxide; OR, odds ratio; CI, confidence interval

The odds ratio and 95% confidence interval in adiposity measures including VAT and SAT was converted by scale to the 100 cm^2^ area

The odds ratio and 95% confidence interval in each air pollutant was scaled to the interquartile range for each pollutant, respectively (11.2 μg/m^3^ for PM_10_, 15.6 ppb for NO_2_, 1.9 ppb for SO_2_, and 0.2 ppm for CO).

Adjusted Model was adjusted for age, smoking status (never-, ex-, or current-smokers), alcohol consumption (never-, ex-, or current-drinkers), and physical activity (yes or no).

### The subgroup results by classifying the BMI, VAT, SAT, and VSR into three levels

**Figure 1:**
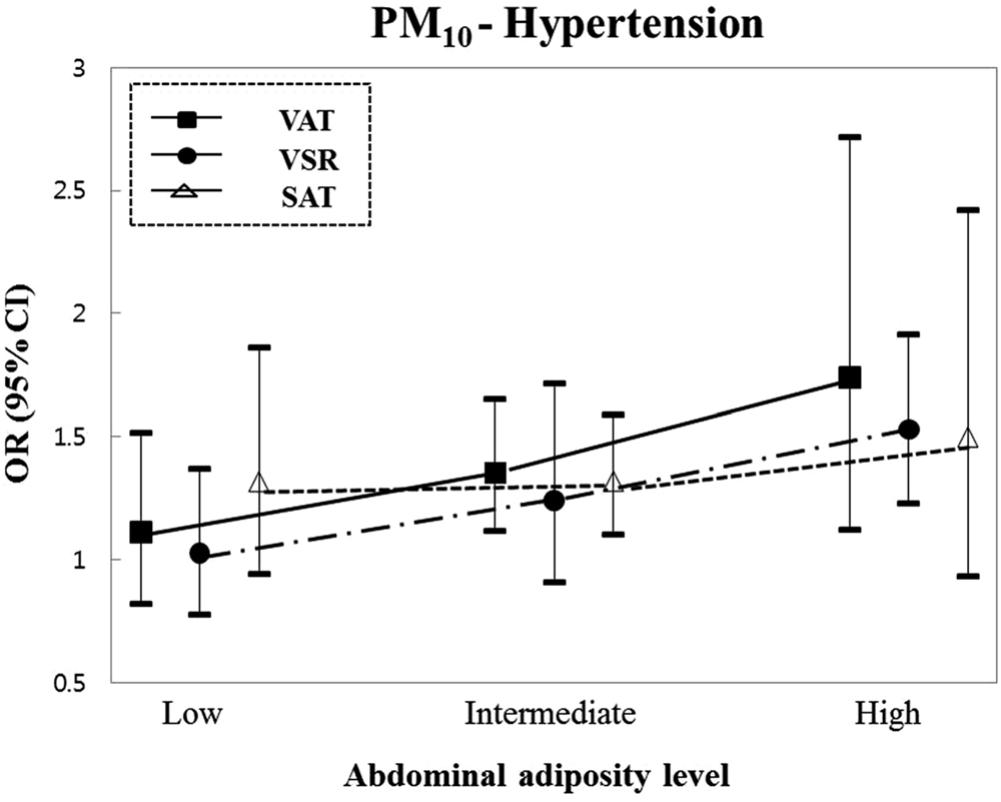
The associations of PM_10_ exposures with hypertension according to VAT, VSR, and SAT categories.

To investigate the association of air pollutants with hypertension based on the degree of each adiposity-related trait, a subgroup analysis was performed by classifying the BMI, VAT, SAT, and VSR into three levels (Table 3 and Fig. 1). In a stratified analysis of BMI, compared with the normal (BMI < 23 kg/m^2^) or overweight group (23 kg/m^2^ ≤ BMI < 25 kg/m^2^), the obese group (BMI ≥ 25 kg/m^2^) showed a positive association between IQR (11.2 μg/m^3^) increase in PM_10_ and hypertension (*OR* = 1.49; 95% *CI* = 1.19–1.88). However, the effect of the BMI modification on PM_10_ and hypertension was not observed (*P*_int_ = 0.1315). Modification effects were observed between ambient PM_10_ and visceral fat-related traits. With regard to VAT, the low adiposity group (VAT ≤ 100 cm^2^) showed no association between PM_10_ and hypertension (*OR* = 1.11; 95% *CI* = 0.82–1.51), whereas the groups with adiposity traits above the intermediate level were positively associated with increased odds of hypertension. In particular, IQR (11.2 μg/m^3^) increase in PM_10_ concentrations in the high-VAT group (VAT > 200 cm^2^) showed the strongest association with hypertension (*OR* = 1.74; 95% *CI* = 1.12–2.71), and the effect of VAT modification was found (*P*_int_ = 0.0197). Likewise, with regard to VSR, the association of PM_10_ with hypertension was the strongest in the high-VSR group (*OR* = 1.53; 95% *CI* = 1.23–1.91), and a modifying effect was also identified (*P*_int_ = 0.0083). However, the effect of SAT modification on PM_10_ and hypertension was not observed (*P*_int_ = 0.4615). We also identified patterns of PM_10_ exposure effects on hypertension by fat distribution, including VAT, SAT, and VSR (Fig. 1). Notably, in the case of VAT and VSR, the association of PM_10_ with hypertension gradually increased with increasing adiposity levels, but there was no difference in the association of PM_10_ and hypertension according to the SAT level. In addition to hypertension, in subgroup results for quantitative BP traits, the association between PM_10_ and SBP or DBP was also differential by adiposity level (Tables S4 and S5). However, unlike hypertension, the association between PM_10_ and increased BP was modified by both VAT and SAT regardless of abdominal fat distribution (all *P*_int_ < 0.05).

**Table 3:** Results of stratified analyses by abdominal adiposity traits for the association between hypertension and exposure to air pollution.

Adiposity	Exposure	Hypertension				*P*_*int*_
		Low adiposity		Intermediate adiposity	High adiposity	
		*OR* (95% *CI*)		*OR* (95% *CI*)	*OR* (95% *CI*)	
BMI (kg/m^2^)	Sample n	BMI < 23 (n = 404)		23 ≤ BMI < 25 (n = 409)	BMI ≥ 25 (n = 604)	
	PM_10_ (μg/m^3^)	1.29 (0.94–1.75)		1.16 (0.87–1.54)	1.49 (1.19–1.88)	0.1315
	NO_2_ (ppb)	1.22 (0.94–1.60)		1.01 (0.76–1.33)	1.09 (0.87–1.36)	0.6692
	SO_2_ (ppb)	1.22 (0.92–1.61)		0.95 (0.74–1.21)	0.96 (0.79–1.17)	0.2756
	CO (ppm)	1.23 (0.90–1.69)		1.41 (1.04–1.90)	1.08 (0.86–1.37)	0.6450
VAT (cm^2^)	Sample n	VAT ≤ 100 (n = 432)		100 < VAT ≤ 200 (n = 803)	VAT > 200 (n = 182)	
	**PM**_**10**_ (**μg/m**^**3**^)	**1**.**11** (**0**.**82–1**.**51**)		**1**.**35** (**1**.**11–1**.**65**)	**1**.**74** (**1**.**12–2**.**71**)	**0**.**0197**
	NO_2_ (ppb)	1.09 (0.83–1.44)		1.15 (0.96–1.39)	0.80 (0.52–1.22)	0.5226
	SO_2_ (ppb)	1.00 (0.78–1.29)		1.03 (0.86–1.22)	0.99 (0.66–1.48)	0.9884
	CO (ppm)	1.37 (1.00–1.88)		1.23 (1.00–1.51)	1.03 (0.71–1.49)	0.4776
SAT (cm^2^)	Sample n	SAT ≤ 100 (n = 344)		100 < SAT ≤ 200 (n = 912)	SAT > 200 (n = 161)	
	PM_10_ (μg/m^3^)	1.32 (0.94–1.85)		1.32 (1.10–1.59)	1.50 (0.93–2.42)	0.4615
	NO_2_ (ppb)	1.23 (0.92–1.65)		1.03 (0.86–1.23)	0.98 (0.62–1.55)	0.3318
	SO_2_ (ppb)	1.06 (0.78–1.44)		0.96 (0.82–1.12)	1.41 (0.86–2.29)	0.5273
	CO (ppm)	1.32 (0.94–1.85)		1.20 (0.99–1.45)	1.04 (0.66–1.63)	0.5571
VSR	Sample n	VSR ≤ 0.8 (n = 460)		0.8 < VSR ≤ 1.0 (n = 318)	VSR > 1.0 (n = 639)	
	**PM**_**10**_ (**μg/m**^**3**^)	**1**.**03** (**0**.**77–1**.**36**)		**1**.**24** (**0**.**90–1**.**71**)	**1**.**53** (**1**.**23–1**.**91**)	**0**.**0083**
	NO_2_ (ppb)	0.95 (0.72–1.24)		1.19 (0.88–1.62)	1.11 (0.90–1.35)	0.3528
	SO_2_ (ppb)	1.04 (0.83–1.32)		1.02 (0.76–1.36)	0.95 (0.78–1.16)	0.4754
	CO (ppm)	1.11 (0.83–1.50)		1.09 (0.77–1.55)	1.31 (1.06–1.63)	0.2411

BMI, body mass index; VAT, visceral adipose tissue; SAT, subcutaneous adipose tissue; VSR, visceral-to-subcutaneous fat ratio; PM_10_, particulate matter ≤ 10 μm in diameter; NO_2_, nitrogen dioxide; SO_2_, sulfur dioxide; CO, carbon monoxide; OR, odds ratio; CI, confidence interval

The odds ratio and 95% confidence interval in each air pollutant was scaled to the interquartile range for each pollutant, respectively (11.2 μg/m^3^ for PM_10_, 15.6 ppb for NO_2_, 1.9 ppb for SO_2_, and 0.2 ppm for CO). The result was adjusted for age, smoking status (never-, ex-, or current-smokers), and alcohol consumption (never-, ex-, or current-drinkers), and physical activity (yes or no). Significant moderation effects are marked in bold (*P*_*int*_ < 0.05).

### The subgroup results stratified by exposure level to PM10

**Figure 2:**
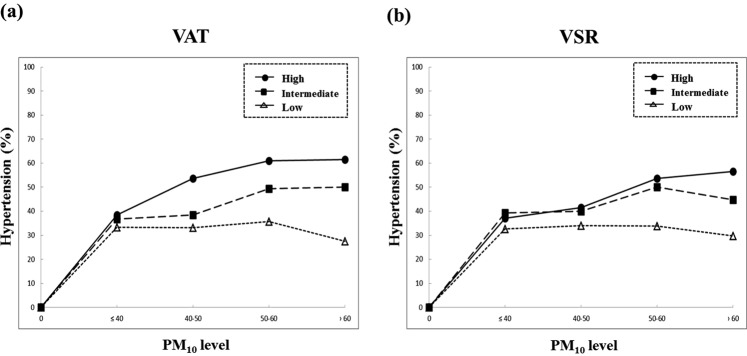
The hypertension (%) according to PM_10_ concentration levels and (**a**) VAT or (**b**) VSR levels.

In addition, we performed the subgroup analysis stratified by PM_10_ levels (Table 4). As shown in Table 4, interestingly, the associations between adiposity and hypertension were also differential by PM_10_ exposure levels. Similar to the results of subgroup analysis stratified by adiposity levels, association between VAT and hypertension was stronger in the high exposure group (*OR* = 3.58; 95% *CI* = 2.32–5.51) than the low exposure group (*OR* = 1.06; 95% *CI* = 0.70–1.61). The pattern of result for VSR was similar to that of VAT. Their modifying effects were also observed (both *P*_int_ < 0.05). In the case of the SAT, the modifying effect was not found (*P*_int_ = 0.1673), although the association with the HTN was greater in the high exposure group (*OR* = 2.52; 95% *CI* = 1.60–3.97) than in the low exposure group (*OR* = 1.63; 95% *CI* = 1.05–2.53). Besides, we indicate the PM_10_ concentration-response (i.e. percentage of hypertension) curves according to the VAT or VSR levels (Fig. 2). As the PM_10_ concentration gradually increased, the proportion of hypertension in the high VAT (Fig. 2a) or VSR (Fig. 2b) group also increased.

**Table 4:** Results of stratified analyses by exposure level to PM_10_ for the association between hypertension and adiposity traits

Adiposity		Hypertension			*P*_*int*_
		Low exposure (n = 362)	Intermediate exposure (n = 702)	High exposure (n = 353)	
		*OR* (95% *CI*)	*OR* (95% *CI*)	*OR* (95% *CI*)	
**BMI** (**kg/m**^**2**^)		**1**.**06** (**0**.**98–1**.**15**)	**1**.**21** (**1**.**14–1**.**28**)	**1**.**25** (**1**.**15–1**.**36**)	**0**.**0088**
**VAT** (**cm**^**2**^)		**1**.**06** (**0**.**70–1**.**61**)	**2**.**06** (**1**.**56–2**.**70**)	**3**.**58** (**2**.**32–5**.**51**)	**<0**.**0001**
SAT (cm^2^)		1.63 (1.05–2.53)	1.77 (1.33–2.37)	2.52 (1.60–3.97)	0.1673
**VSR**		**0**.**56** (**0**.**31–1**.**02**)	**1**.**43** (**0**.**98–2**.**09**)	**2**.**24** (**1**.**30–3**.**89**)	**0**.**0008**

BMI, body mass index; VAT, visceral adipose tissue; SAT, subcutaneous adipose tissue; VSR, visceral-to-subcutaneous fat ratio; OR, odds ratio; CI, confidence interval.

The odds ratio and 95% confidence interval in adiposity measures including VAT and SAT was converted by scale to the 100 cm^2^ area.

The result was adjusted for age, smoking status (never-, ex-, or current-smokers), and alcohol consumption (never-, ex-, or current-drinkers), and physical activity (yes or no).

Significant modifying effect of PM_10_ level on adiposity traits and hypertension are marked in bold (*P*_*int*_ < 0.05).

## Discussion

We investigated the associations of ambient air pollution and hypertension in adult Korean men and whether these associations were differently modified by each fat depot, including VAT and SAT. In the subgroup analysis classified according to the level of visceral abdominal fat, we observed that the associations between PM_10_ concentration and hypertension were much stronger in the high-VAT group than in the low-VAT group. Its modification effect was also identified. Besides, the results of the subgroup analysis for VSR showed a pattern similar to that for VAT. In contrast, there was no big difference in the associations between PM_10_ concentration and hypertension in the subgroup of SAT levels. Our findings suggest that the positive association of PM_10_ exposure with hypertension is more apparent in people with greater visceral fat rather than in people with subcutaneous fat.

Visceral adiposity has been associated with the occurrence of metabolic and cardiovascular diseases^24,25^. With regard to hypertension, such relations are especially prominent in the Asian population. Hayashi *et al*. found a significant association between visceral adiposity and hypertension in Japanese Americans^26,27^. In 2011, Koh *et al*. also reported a similar association, indicating that visceral adiposity, rather than subcutaneous fat, is closely related to high BP in Japanese men^28^. On the contrary, an epidemiological study conducted in a group of African Americans and Hispanic Americans observed no significant relationship between visceral adiposity and hypertension among men^29^. The effect of visceral fat on hypertension in Asian populations may be more important, as Asians have a greater deposition of visceral fat compared with other ethnic populations. Our results showed that VAT had the highest risk ratio for hypertension, and VSR also showed a significant association with hypertension. These results support the importance of visceral fat in the Asian population.

Recent epidemiological studies have identified the modification effects of obesity defined by BMI on increased BP and ambient air pollution. In 2013, Zhao *et al*. investigated whether the association between airborne pollutants and increased BP or hypertension is modified by obesity status in a Chinese population^9^. They reported that the risks for high BP and hypertension by ambient air pollution were greater in overweight/obese adult men. Similarly, more recent study conducted in Chinese children identified significant interaction effects of obesity and long-term air pollution exposure on BP and hypertension^10^. Our results also showed that the association of PM_10_ with hypertension is stronger in the obese group than in the non-obese (normal or overweight) group, even though its modification effect was not considered significant. Rather, the modification effect was more pronounced in the abdominal visceral fat-related traits measured directly by CT, than in the BMI, an indirect measure of body fat. These findings highlight the importance of using accurate fat distribution indicators measured directly by CT to better understand the epidemiological or biological association between air pollution, hypertension, and obesity.

The physiological mechanism for the difference in the association between PM_10_ exposure and hypertension by visceral fat levels is not clear. However, several plausible hypotheses associated with adipocyte have been proposed. The most likely mechanism is inflammation. Prolonged exposure to PM causes hypertension-related vascular endothelial dysfunction via local vascular inflammation or systemic inflammation^30–33^. Such endothelial dysfunction may be involved in an increased systemic vascular resistance, thereby leading to the development of hypertension^34^. Similarly, excess visceral fat accelerates systemic inflammation by releasing inflammatory adipokines, such as TNF-α, IL-6, C-reactive protein, resistin, and macrophage chemoattractant protein-1, in obese humans^35,36^. Besides, the level of adiponectin associated with anti-inflammatory function is inversely correlated with visceral adiposity. Both PM and visceral fat accumulation are closely associated with increased inflammation response, and the strong association of PM_10_ and hypertension in the high-visceral adiposity group may be explained by the combined effects of visceral fat and ambient air pollution on vascular and/or systemic inflammation. Another hypothesis is an increase in oxidative stress. Oxidative stress response is one of the major mechanisms underlying hypertension^37^. Particles in air pollutants alter the vasomotor balance by inducing the production of ROS in the vascular endothelium. Similarly, obesity is involved in systemic oxidative stress responses, and ROS is known to be more associated with visceral fat accumulation than subcutaneous fat accumulation^38^. In addition, both PM and visceral fat contribute to the imbalance in the autonomic nervous system function^30,39^, which can lead to the development of hypertension^40^.

We used four components (i.e. PM_10_, NO_2_, SO_2_, and CO) of ambient air pollution with partially different characteristics. Contrary to the PM_10_, the gaseous pollutants, such as NO_2_, SO_2_, and CO, were not associated with hypertension in our data. Similarly, several previous studies have shown that PM compared to gaseous substances has a strong negative effect on health outcomes^4,41,42^. PM is a complex mixture of different particles (solid and liquid particles) with physical, chemical and toxicological properties, unlike gaseous pollutants derived from a specific gas^43^. Such PM in comparison with gaseous pollutants is responsible for a large portion of the pathogenic effects, leading to the development of systemic pro-inflammation via activation of innate immunity as well as enhancement of free radical reactions in cells and tissues^43,44^. The larger association of PM_10_ than gaseous pollutants may be explained by the different nature of the air pollutants.

In this study, we measured VAT and SAT using CT to determine the accurate quantitative measurement of fat mass. To our knowledge, we found for the first time that the relationship between PM_10_ and prevalence of hypertension was more strongly associated with abdominal visceral fat accumulation than subcutaneous fat accumulation. However, several limitations need to be discussed. First, our study had a cross-sectional design, which cannot be used to determine the causal relationship between ambient air pollution, adiposity traits, and hypertension. Second, this study did not include women, due to the large differences in abdominal fat distribution by sex. Air pollution- or adiposity-induced health outcome may differ by gender due to activity patterns, sex hormones, occupational exposure, and lifestyle, in addition to differences in fat distribution. Therefore, our main results, including only adult men, can be difficult to generalize in women. Third, we could not consider socio-economic status information as a confounding factor, due to the absence of relevant data. Therefore, the results may be likely to be affected by residual bias. In addition, it was difficult to estimate a longer period of exposure concentration, because we do not have any information regarding the residential history of the subjects. Therefore, we finally used only the annual average air pollutant concentrations of the year of participant’s medical checkup. Lastly, to estimate air pollution exposure concentrations, we used the community-level exposure assessment which can reduce variations in exposure using a zip code instead of an individual’s exact exposure estimate because of lack of relevant data. This way does not reflect various factors such as indoor or occupational exposure level, diversity of mobility among individuals, residence history, and proximity to major roads. Thus, this may have the potential for exposure misclassification.

In conclusion, we identified that association between PM_10_ and hypertension in Asian men is different by abdominal fat, especially visceral fat levels. However, more work studying in women or other populations is needed to understand the association between obesity, air pollution exposure, and hypertension.

## Supplementary information

Supplymentary Information
