# Supplementary material for: Effects of abdominal visceral fat compared with those of subcutaneous fat on the association between PM10 and hypertension in Korean men: A cross-sectional study
Source: Sci Rep. 2019 Apr 11;9:5951. doi: 10.1038/s41598-019-42398-1 (PMC6459915; doi:10.1038/s41598-019-42398-1)
Supplement: Supplementary file 1 — Supplymentary Information [file 41598_2019_42398_MOESM1_ESM.docx]

**[Title page]**

**Effects of abdominal visceral fat compared with those of subcutaneous fat on the association between PM_10_ and hypertension in Korean men: A cross-sectional study**

Hyun-Jin Kim^1^, Hyuktae Kwon^2^, Su-Min Jeong^2^, Seo Eun Hwang^2^, Jin-Ho Park^2,3^

^1^National Cancer Control Institute, National Cancer Center, Goyang 10408, South Korea

^2^Department of Family Medicine, Seoul National University Hospital, Seoul 03080, South Korea

^3^Department of Family Medicine, Seoul National University College of Medicine, Seoul 03080, South Korea

**†Correspondence: Jin-Ho Park, M.D., Ph.D. Professor**

Department of Family Medicine, Seoul National University, Hospital, Seoul National University College of Medicine, 103 Daehakro, Yeongun-dong, Jongno-gu, Seoul, 03080, Korea. Tel: +82. 2-2072-0865, Fax: +82. 2-766-3276, E-mail: pjhn@snu.ac.kr

Table S1. The results of a univariate analysis

|  |  | Hypertension | | |  |
| --- | --- | --- | --- | --- | --- |
|  |  | No |  | Yes |  |
| Characteristics |  | n(%) or mean (SD) |  | n(%) or mean (SD) | *P* |
| n |  | 824 (58.1) |  | 593 (41.9) |  |
| Age (years) |  | 54.5 (9.4) |  | 57.9 (8.7) | <0.0001 |
| Smoking |  |  |  |  | 0.0101 |
| Never |  | 180 (21.8) |  | 129 (21.7) |  |
| Former-smokers |  | 351 (42.6) |  | 313 (52.8) |  |
| Current-smokers |  | 293 (35.6) |  | 151 (25.5) |  |
| Alcohol drinking |  |  |  |  | 0.2132 |
| Never |  | 210 (26.5) |  | 136 (22.9) |  |
| Former-drinkers |  | 66 (8.0) |  | 55 (9.3) |  |
| Current- drinkers |  | 540 (65.5) |  | 402 (67.8) |  |
| Physical activity |  |  |  |  | 0.1602 |
| Yes |  | 322 (39.1) |  | 210 (35.4) |  |
| No |  | 502 (60.9) |  | 383 (64.6) |  |
| BMI(kg/m^2^) |  | 24.1 (2.8) |  | 25.2 (3.0) | <0.0001 |

BMI, body mass index

Data are presented as mean (standard deviation) for continuous variables, or n (%) for categorical variables.

Table S2. Air pollutions (annual average concentrations) and their distributions

|  | Mean | SD | IQR | Pearson’s correlation coefficients | | | |
| --- | --- | --- | --- | --- | --- | --- | --- |
|  |  |  |  | PM_10_ | NO_2_ | SO_2_ | CO |
| Distance between monitoring stations and home | | | | | | | |
| distance (km) | 4.7 | 7.6 | 2.47 | - | - | - | - |
| Air pollutants | | | | | | | |
| PM_10_ (μg/m^3^) | 49.7 | 8.4 | 11.2 | 1 | 0.32^***^ | 0.21^***^ | 0.30^***^ |
| NO_2_ (ppb) | 29.0 | 12.0 | 15.6 | - | 1 | 0.36^***^ | 0.61^***^ |
| SO_2_ (ppb) | 5.2 | 1.6 | 1.9 | - | - | 1 | 0.34^***^ |
| CO (ppm) | 0.56 | 0.14 | 0.2 | - | - | - | 1 |

SD, standard deviation; IQR, interquartile range; PM_10_, particulate matter ≤ 10 μm in diameter; NO_2_, nitrogen dioxide; SO_2_, sulfur dioxide; CO, carbon monoxide.

^*^*P* < 0.05, ^**^*P* < 0.01, ^***^*P* < 0.001

Table S3. Linear regression results for the association between air pollution, adiposity-related traits, and systolic or diastolic blood pressure

|  | SBP | | | | |  | DBP | | | | |
| --- | --- | --- | --- | --- | --- | --- | --- | --- | --- | --- | --- |
|  | Unadjusted Model | |  | Adjusted Model | |  | Unadjusted Model | |  | Adjusted Model | |
|  | *β (SE)* | *P* |  | *β (SE)* | *P* |  | *β (SE)* | *P* |  | *β (SE)* | *P* |
| Adiposity trait |  |  |  |  |  |  |  |  |  |  |  |
| BMI (kg/m^2^) | 1.17 (0.14) | <0.0001 |  | 1.25 (0.14) | <0.0001 |  | 0.75 (0.10) | <0.0001 |  | 0.75 (0.10) | <0.0001 |
| VAT (cm^2^) | 6.21 (0.70) | <0.0001 |  | 5.91 (0.70) | <0.0001 |  | 3.67 (0.50) | <0.0001 |  | 3.63 (0.50) | <0.0001 |
| SAT (cm^2^) | 4.86 (0.76) | <0.0001 |  | 5.34 (0.76) | <0.0001 |  | 3.27 (0.53) | <0.0001 |  | 3.26 (0.54) | <0.0001 |
| VSR | 3.23 (1.02) | 0.0015 |  | 2.30 (1.04) | 0.0268 |  | 1.55 (0.71) | 0.0295 |  | 1.57 (0.73) | 0.0315 |
| Air pollution |  |  |  |  |  |  |  |  |  |  |  |
| PM_10_ (μg/m^3^) | 1.94 (0.57) | 0.0006 |  | 2.13 (0.58) | 0.0002 |  | 1.36 (0.39) | 0.0006 |  | 1.33 (0.41) | 0.0010 |
| NO_2_ (ppb) | 1.14 (0.56) | 0.0410 |  | 1.16 (0.55) | 0.0358 |  | 0.51 (0.39) | 0.1902 |  | 0.55 (0.39) | 0.1577 |
| SO_2_ (ppb) | 0.10 (0.52) | 0.8390 |  | 0.16 (0.51) | 0.7587 |  | 0.24 (0.36) | 0.4985 |  | 0.22 (0.36) | 0.5491 |
| CO (ppm) | 1.25 (0.60) | 0.0378 |  | 1.26 (0.60) | 0.0354 |  | 0.63 (0.42) | 0.1340 |  | 0.62 (0.42) | 0.1397 |

BMI, Body mass index; VAT, visceral adipose tissue; SAT, subcutaneous adipose tissue; VSR, visceral-to-subcutaneous fat ratio; PM_10_, particulate matter ≤ 10 μm in diameter; NO_2_, nitrogen dioxide; SO_2_, sulfur dioxide; CO, carbon monoxide; SE, standard error; SBP, systolic blood pressure; DBP, diastolic blood pressure

The beta coefficient and standard error in adiposity measures including VAT and SAT was converted by scale to the 100 cm^2^ area

The beta coefficient and standard error in each air pollutant was scaled to the interquartile range for each pollutant, respectively (11.2 μg/m^3^ for PM_10_, 15.6 ppb for NO_2_, 1.9 ppb for SO_2_, and 0.2 ppm for CO).

Adjusted Model was adjusted for age, smoking status (never-, ex-, or current-smokers), alcohol consumption (never-, ex-, or current-drinkers), and physical activity (yes or no).

Table S4. Linear regression results stratified by abdominal adiposity traits for the association between systolic blood pressure and exposure to air pollution

|  |  |  |  | SBP | | | | | | |  |  |
| --- | --- | --- | --- | --- | --- | --- | --- | --- | --- | --- | --- | --- |
|  |  | Low adiposity | | | |  | Intermediate adiposity | |  | High adiposity | |  |
| Adiposity | Exposure | *β (SE)* | | | *P* |  | *β (SE)* | *P* |  | *β (SE)* | *P* | *P_int_* |
| BMI (kg/m^2^) | Sample n | BMI < 23 (n = 404) | | | |  | 23 ≤ BMI < 25 (n = 409) | |  | BMI ≥ 25 (n = 604) | |  |
|  | PM_10_ (μg/m^3^) | 0.11 (1.10) | | | 0.9167 |  | 2.13 (1.06) | 0.0450 |  | 3.41 (0.85) | <0.0001 | 0.0080 |
|  | NO_2_ (ppb) | 1.06 (0.95) | | | 0.2674 |  | 0.89 (1.05) | 0.3961 |  | 1.77 (0.86) | 0.0387 | 0.5190 |
|  | SO_2_ (ppb) | 1.26 (0.98) | | | 0.1980 |  | -0.49 (0.91) | 0.5914 |  | -0.08 (0.77) | 0.9204 | 0.4535 |
|  | CO (ppm) | -0.14 (1.12) | | | 0.9012 |  | 1.81 (1.10) | 0.0991 |  | 1.66 (0.90) | 0.0643 | 0.1786 |
|  |  |  | | |  |  |  |  |  |  |  |  |
| VAT (cm^2^) | Sample n | VAT ≤ 100 (n = 432) | | | |  | 100 < VAT ≤ 200 (n = 803) | |  | VAT > 200 (n = 182) | |  |
|  | PM_10_ (μg/m^3^) | 0.05 (1.07) | | | 0.9663 |  | 2.71 (0.75) | 0.0003 |  | 4.42 (1.48) | 0.0031 | 0.0024 |
|  | NO_2_ (ppb) | 0.74 (0.97) | | | 0.4497 |  | 1.88 (0.72) | 0.0096 |  | -1.30 (1.52) | 0.3917 | 0.8297 |
|  | SO_2_ (ppb) | 0.40 (0.88) | | | 0.6453 |  | 0.05 (0.69) | 0.9430 |  | 0.42 (1.46) | 0.7739 | 0.9693 |
|  | CO (ppm) | 0.10 (1.10) | | | 0.9243 |  | 1.88 (0.80) | 0.0202 |  | 1.09 (1.36) | 0.4217 | 0.2829 |
|  |  |  | | |  |  |  |  |  |  |  |  |
| SAT (cm^2^) | Sample n | SAT ≤ 100 (n = 344) | | | |  | 100 < SAT ≤ 200 (n = 912) | |  | SAT > 200 (n = 161) | |  |
|  | PM_10_ (μg/m^3^) | -0.33 (1.17) | | | 0.7793 |  | 2.63 (0.70) | 0.0002 |  | 4.93 (1.76) | 0.0058 | 0.0011 |
|  | NO_2_ (ppb) | 0.87 (1.02) | | | 0.3909 |  | 1.24 (0.69) | 0.0720 |  | 0.02 (1.77) | 0.9908 | 0.9468 |
|  | SO_2_ (ppb) | 0.10 (1.04) | | | 0.9207 |  | -0.10 (0.61) | 0.8756 |  | 2.13 (1.84) | 0.2479 | 0.3666 |
|  | CO (ppm) | 1.30 (1.17) | | | 0.2670 |  | 1.13 (0.74) | 0.1268 |  | 1.20 (1.75) | 0.4929 | 0.8300 |
|  |  |  | | |  |  |  |  |  |  |  |  |
| VSR | Sample n | VSR ≤ 0.8 (n = 460) | | | |  | 0.8 < VSR ≤ 1.0 (n = 318) | |  | VSR > 1.0 (n = 639) | |  |
|  | PM_10_ (μg/m^3^) | 1.65 (1.05) | | | 0.1175 |  | 1.26 (1.21) | 0.2962 |  | 2.60 (0.84) | 0.0021 | 0.4063 |
|  | NO_2_ (ppb) | 2.08 (1.00) | | | 0.0381 |  | 0.10 (1.16) | 0.9345 |  | 1.05 (0.80) | 0.1883 | 0.5972 |
|  | SO_2_ (ppb) | 0.54 (0.88) | | | 0.5368 |  | 0.71 (1.11) | 0.5192 |  | -0.65 (0.77) | 0.4014 | 0.1819 |
|  | CO (ppm) | 1.75 (1.11) | | | 0.1167 |  | -0.85 (1.34) | 0.5254 |  | 1.86 (0.83) | 0.0256 | 0.6636 |

BMI, body mass index; VAT, visceral adipose tissue; SAT, subcutaneous adipose tissue; VSR, visceral-to-subcutaneous fat ratio; PM_10_, particulate matter ≤ 10 μm in diameter; NO_2_, nitrogen dioxide; SO_2_, sulfur dioxide; CO, carbon monoxide; SE, standard error; SBP, systolic blood pressure

The beta coefficient and standard error in each air pollutant was scaled to the interquartile range for each pollutant, respectively (11.2 μg/m^3^ for PM_10_, 15.6 ppb for NO_2_, 1.9 ppb for SO_2_, and 0.2 ppm for CO). The result was adjusted for age, smoking status (never-, ex-, or current-smokers), and alcohol consumption (never-, ex-, or current-drinkers), and physical activity (yes or no).

Table S5. Linear regression results stratified by abdominal adiposity traits for the association between diastolic blood pressure and exposure to air pollution

|  |  |  |  | DBP | | | | | | |  |  |
| --- | --- | --- | --- | --- | --- | --- | --- | --- | --- | --- | --- | --- |
|  |  | Low adiposity | | | |  | Intermediate adiposity | |  | High adiposity | |  |
| Adiposity | Exposure | *β (SE)* | | | *P* |  | *β (SE)* | *P* |  | *β (SE)* | *P* | *P_int_* |
| BMI (kg/m^2^) | Sample n | BMI < 23 (n = 404) | | | |  | 23 ≤ BMI < 25 (n = 409) | |  | BMI ≥ 25 (n = 604) | |  |
|  | PM_10_ (μg/m^3^) | 0.05 (0.77) | | | 0.9397 |  | 1.86 (0.76) | 0.0142 |  | 1.75 (0.60) | 0.0036 | 0.0722 |
|  | NO_2_ (ppb) | 0.62 (0.67) | | | 0.3556 |  | 0.68 (0.75) | 0.3656 |  | 0.61 (0.60) | 0.3093 | 0.9924 |
|  | SO_2_ (ppb) | 1.08 (0.69) | | | 0.1174 |  | 0.00 (0.65) | 0.9951 |  | -0.21 (0.54) | 0.7021 | 0.2160 |
|  | CO (ppm) | -0.48 (0.79) | | | 0.5392 |  | 1.44 (0.78) | 0.0672 |  | 0.71 (0.63) | 0.2597 | 0.2726 |
|  |  |  | | |  |  |  |  |  |  |  |  |
| VAT (cm^2^) | Sample n | VAT ≤ 100 (n = 432) | | | |  | 100 < VAT ≤ 200 (n = 803) | |  | VAT > 200 (n = 182) | |  |
|  | PM_10_ (μg/m^3^) | -0.49 (0.75) | | | 0.5152 |  | 1.86 (0.52) | 0.0004 |  | 2.64 (1.09) | 0.0168 | 0.0019 |
|  | NO_2_ (ppb) | 0.11 (0.69) | | | 0.8690 |  | 1.18 (0.50) | 0.0198 |  | -1.60 (1.11) | 0.1521 | 0.6142 |
|  | SO_2_ (ppb) | 0.55 (0.62) | | | 0.3723 |  | 0.06 (0.48) | 0.9028 |  | 0.36 (1.07) | 0.7351 | 0.7907 |
|  | CO (ppm) | -0.33 (0.78) | | | 0.6730 |  | 1.27 (0.56) | 0.0249 |  | -0.09 (1.00) | 0.9270 | 0.5140 |
|  |  |  | | |  |  |  |  |  |  |  |  |
| SAT (cm^2^) | Sample n | SAT ≤ 100 (n = 344) | | | |  | 100 < SAT ≤ 200 (n = 912) | |  | SAT > 200 (n = 161) | |  |
|  | PM_10_ (μg/m^3^) | -1.09 (0.82) | | | 0.1839 |  | 1.95 (0.49) | <0.0001 |  | 3.01 (1.29) | 0.0210 | 0.0007 |
|  | NO_2_ (ppb) | 0.54 (0.72) | | | 0.4544 |  | 0.62 (0.48) | 0.1996 |  | -0.77 (1.29) | 0.5479 | 0.5089 |
|  | SO_2_ (ppb) | 0.48 (0.74) | | | 0.5119 |  | -0.01 (0.43) | 0.9780 |  | 1.20 (1.33) | 0.3679 | 0.8358 |
|  | CO (ppm) | 0.34 (0.82) | | | 0.6799 |  | 0.68 (0.52) | 0.1924 |  | 0.41 (1.27) | 0.7490 | 0.7779 |
|  |  |  | | |  |  |  |  |  |  |  |  |
| VSR | Sample n | VSR ≤ 0.8 (n = 460) | | | |  | 0.8 < VSR ≤ 1.0 (n = 318) | |  | VSR > 1.0 (n = 639) | |  |
|  | PM_10_ (μg/m^3^) | 1.05 (0.76) | | | 0.1675 |  | 0.08 (0.82) | 0.9259 |  | 1.95 (0.58) | 0.0009 | 0.2597 |
|  | NO_2_ (ppb) | 0.68 (0.73) | | | 0.3507 |  | 0.02 (0.79) | 0.9840 |  | 0.72 (0.56) | 0.1926 | 0.8239 |
|  | SO_2_ (ppb) | 0.67 (0.63) | | | 0.2895 |  | -0.09 (0.75) | 0.9092 |  | -0.11 (0.54) | 0.8310 | 0.2415 |
|  | CO (ppm) | 0.35 (0.81) | | | 0.6654 |  | -0.55 (0.91) | 0.5469 |  | 1.30 (0.58) | 0.0252 | 0.2278 |

BMI, body mass index; VAT, visceral adipose tissue; SAT, subcutaneous adipose tissue; VSR, visceral-to-subcutaneous fat ratio; PM_10_, particulate matter ≤ 10 μm in diameter; NO_2_, nitrogen dioxide; SO_2_, sulfur dioxide; CO, carbon monoxide; SE, standard error; DBP, diastolic blood pressure

The beta coefficient and standard error in each air pollutant was scaled to the interquartile range for each pollutant, respectively (11.2 μg/m^3^ for PM_10_, 15.6 ppb for NO_2_, 1.9 ppb for SO_2_, and 0.2 ppm for CO). The result was adjusted for age, smoking status (never-, ex-, or current-smokers), and alcohol consumption (never-, ex-, or current-drinkers), and physical activity (yes or no).
